# Impact of Chemical Fluctuations on Stacking Fault Energies of CrCoNi and CrMnFeCoNi High Entropy Alloys from First Principles

**DOI:** 10.3390/e20090655

**Published:** 2018-08-30

**Authors:** Yuji Ikeda, Fritz Körmann, Isao Tanaka, Jörg Neugebauer

**Affiliations:** 1 Computational Materials Design, Max-Planck-Institut für Eisenforschung GmbH, 40237 Düsseldorf, Germany; 2 Materials Science and Engineering, Kyoto University, Kyoto 606-8501, Japan; 3 Materials Science and Engineering, Delft University of Technology, 2628 CD Delft, The Netherlands; 4 Center for Elements Strategy Initiative for Structure Materials (ESISM), Kyoto University, Kyoto 606-8501, Japan; 5 Center for Materials Research by Information Integration, National Institute for Materials Science (NIMS), Tsukuba 305-0047, Japan; 6 Nanostructures Research Laboratory, Japan Fine Ceramics Center, Nagoya 456-8587, Japan

**Keywords:** high-entropy alloy, stacking-fault energy, density functional theory

## Abstract

Medium and high entropy alloys (MEAs and HEAs) based on 3d transition metals, such as face-centered cubic (fcc) CrCoNi and CrMnFeCoNi alloys, reveal remarkable mechanical properties. The stacking fault energy (SFE) is one of the key ingredients that controls the underlying deformation mechanism and hence the mechanical performance of materials. Previous experiments and simulations have therefore been devoted to determining the SFEs of various MEAs and HEAs. The impact of local chemical environment in the vicinity of the stacking faults is, however, still not fully understood. In this work, we investigate the impact of the compositional fluctuations in the vicinity of stacking faults for two prototype fcc MEAs and HEAs, namely CrCoNi and CrMnFeCoNi by employing first-principles calculations. Depending on the chemical composition close to the stacking fault, the intrinsic SFEs vary in the range of more than 150 mJ/m${}^{2}$ for both the alloys, which indicates the presence of a strong driving force to promote particular types of chemical segregations towards the intrinsic stacking faults in MEAs and HEAs. Furthermore, the dependence of the intrinsic SFEs on local chemical fluctuations reveals a highly non-linear behavior, resulting in a non-trivial interplay of local chemical fluctuations and SFEs. This sheds new light on the importance of controlling chemical fluctuations via tuning, e.g., the annealing condition to obtain the desired mechanical properties for MEAs and HEAs.

## 1. Introduction

High entropy alloys (HEAs) or complex concentrated alloys (CCAs) based on 3d transition metals have attracted enormous attention recently, in particular due to their outstanding mechanical properties. The equiatomic CrMnFeCoNi HEA, also often termed the Cantor alloy [1], has an excellent combination of strength and ductility [2,3,4,5]. Different strategies have been proposed to further improve the mechanical properties, e.g., by tuning the chemical compositions of CrMnFeCo and CrMnFeCoNi towards nonequiatomic alloys [6,7,8,9,10] or by resorting to so-called medium entropy alloys (MEAs), such as CrCoNi alloys [11,12,13].

A key factor in controlling the underlying deformation mechanism and therewith tuning the mechanical properties is the stacking fault energy (SFE). Low SFEs can induce, e.g., transformation- induced plasticity (TRIP) or twinning-induced plasticity (TWIP) [14,15,16], and for this reason, SFEs of HEAs and CCAs have been investigated previously in numerous experimental [13,17,18,19] as well as theoretical studies [20,21,22,23,24,25,26,27,28,29,30,31]. Interestingly, in a recent experimental work [17], the measured SFEs for equiatomic CrMnFeCoNi revealed large fluctuations, and it was proposed that the SFEs of CrMnFeCoNi may sensitively depend on the local chemical environments in the vicinity of the stacking faults (SFs). Also, in computational works [24,26,29,30], large fluctuations of SFEs have been found for MEAs and HEAs based on 3d transition metals, like CrCoNi and CrMnFeCoNi, which indicates a strong dependence of the SFEs on local chemical fluctuations close to the SFs. A recent experimental work [32] revealed that chemical inhomogeneity in a Cr${}_{10}$Mn${}_{30}$Fe${}_{50}$Co${}_{10}$-based alloy also caused a large deterioration of mechanical properties. Although these results suggest an important role of chemical fluctuations in these alloys, the impact of such fluctuations on the SFEs of MEAs and HEAs has not been intensively investigated yet. In a recent study employing first-principles calculations [24], it was proposed that the SFEs of CrCoNi and CrCoFeNi may depend on the valence electron concentration (VEC) of the elements near the SF. In that study, however, only a very limited number of configurations and local compositions near the SF were evaluated, prohibiting a further quantitative analysis.

In the present study, we comprehensively investigate the impact of compositional fluctuations near the intrinsic SF (ISF) on the intrinsic SFE (ISFE) for the face-centered cubic (fcc) equiatomic CrCoNi and CrMnFeCoNi alloys based on first-principles calculations. The ISFEs were calculated using supercells with and without an ISF, while the chemical disorder was modeled using the coherent potential approximation (CPA) [33,34,35]. The combination of supercells and the CPA makes it possible to systematically investigate arbitrary local composition ratios in the vicinity of the ISFs and to elucidate the impact of compositional fluctuations for individual elements using relatively small, and thus computationally efficient, supercells.

## 2. Computational Details

**Figure 1:**
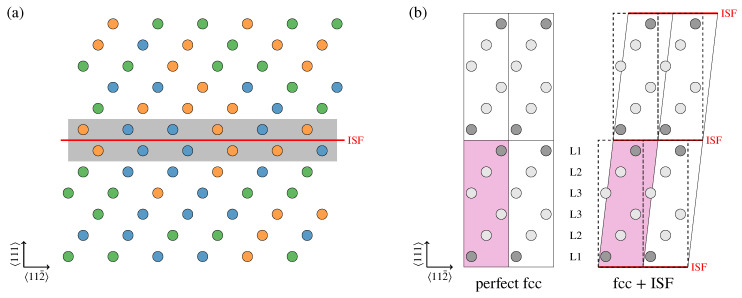
(**a**) Schematic of the intrinsic stacking faults (ISF) in a three-component face-centered cubic (fcc) equiatomic disordered alloy. The red line indicates the ISF. The circles represent atoms, which are colored differently according to the chemical elements. Notice that local chemical fluctuations can exist close to the ISF (gray background region) even if, on average, the alloy has an equiatomic composition; (**b**) Projected atomic positions of the simulation cells with and without ISFs. The red background regions represent the six-layer simulation cells, while the dashed boxes indicate the cell shape of the perfect fcc structure. The red lines indicate the ISFs. The circles represent atoms which are colored differently according to the given mixing ratios of the constitutive chemical elements in the CPA to model the compositional fluctuations. The atomic layers are labeled L1, L2, and L3 according to the distance from the ISFs.

We investigate the impact of the local chemical environment in the vicinity of ISFs on the ISFEs, as described in Figure 1a. In principle, the impact of the local chemical environment can be investigated by employing large supercells. As this approach turns out to be computationally too demanding for systematically screening a large number of different compositional fluctuations near the ISFE, we resort to an alternative approach. To reduce the computational cost while keeping the key physical ingredients, we combined the CPA with the supercell approach. Specifically, the impact of the local chemical environment near the ISFs on the ISFEs was investigated using six-layer supercells with and without an ISF as shown in Figure 1b, and the compositional fluctuation in the vicinity of the ISF was introduced based on the CPA by modifying the mixing ratios of the chemical elements in the L1 layers close to the ISF (see Figure 1b) from the equiatomic while keeping the equiatomic ratios in the subsequent L2 and L3 layers. The ISF was introduced by tilting the 〈111〉 axis of the perfect-fcc simulation cell by $6\langle 11\bar{2}\rangle /a$, where *a* is the fcc lattice constant [36]. The ISFEs, $\gamma_{\mathrm{ISF}}$, were computed with

(1) $$\begin{array}{cc} \gamma_{\mathrm{ISF}} & =\frac{E^{\mathrm{fcc}+\mathrm{ISF}}-E^{\mathrm{fcc}}}{A}, \end{array}$$

where $E^{\mathrm{fcc}+\mathrm{ISF}}$ and $E^{\mathrm{fcc}}$ are the energies of the simulation cells with and without ISFs, respectively, and *A* denotes the area of the ISFs. As we focused on the impacts of the chemical fluctuations near the ISFs, the computed ISFEs are shown as the differences from the ones without compositional fluctuations. The impacts of lattice vibrations [26,29], magnetic excitations [21], chemical short-range order (SRO) [30], and volume changes [9] on the ISFEs have been investigated previously and were therefore not included in the present study. We also note that local lattice distortions, which cannot be considered in the CPA, possibly affect the ISFEs.

SFEs are also often computed based on the axial Ising model [37], in which the SFE is derived from the energy differences among the fcc, hexagonal close-packed (hcp), and sometimes double hcp phases. By construction, however, the axial Ising model cannot capture the impact of local chemical fluctuations close to the SFs, because the perfect fcc and hcp structures do not include the SFs explicitly. The presently employed supercell approach, in contrast, enabled us to explicitly investigate the impacts of local chemical fluctuations close to the SFs.

The electronic structure calculations were performed with the exact-muffin-tin-orbital (EMTO) method [38,39,40,41,42] in combination with the full-charge-density (FCD) method [43,44] within the density functional theory (DFT) framework. The DFT energies were calculated within the generalized gradient approximation (GGA) of the Perdew–Burke–Ernzerhof (PBE) form [45] in the following perturbative manner [46]. The electronic densities were first calculated using the local-density approximation (LDA), and then the total energies were calculated within the GGA-PBE via the FCD method based on the obtained electronic densities. This approach makes the calculations faster and often more stable while keeping the accuracy of the GGA [46], and it has therefore been employed in numerous studies using the EMTO approach [47,48,49,50,51,52,53,54,55,56]. The Brillouin zones were sampled by 22×22×4*k*-point meshes per six-atom computational unit cell, where the 〈111〉 direction in Figure 1b was set to be the *z*-axis. As experiments [57] and first-principles calculations [52,53] have revealed that both CrCoNi and CrMnFeCoNi are paramagnetic (PM) at room temperature, we simulated the magnetic disorder with random magnetic moments by employing the disordered local moment (DLM) model [42,58,59] in combination with the CPA. The lattice constant was fixed to 3.56 Å for CrCoNi and 3.6 Å for CrMnFeCoNi, which are close to the experimental values [1,60,61,62,63,64], and the atomic positions were fixed to keep the rigid-sphere packing.

## 3. Results and Discussion

**Figure 2:**
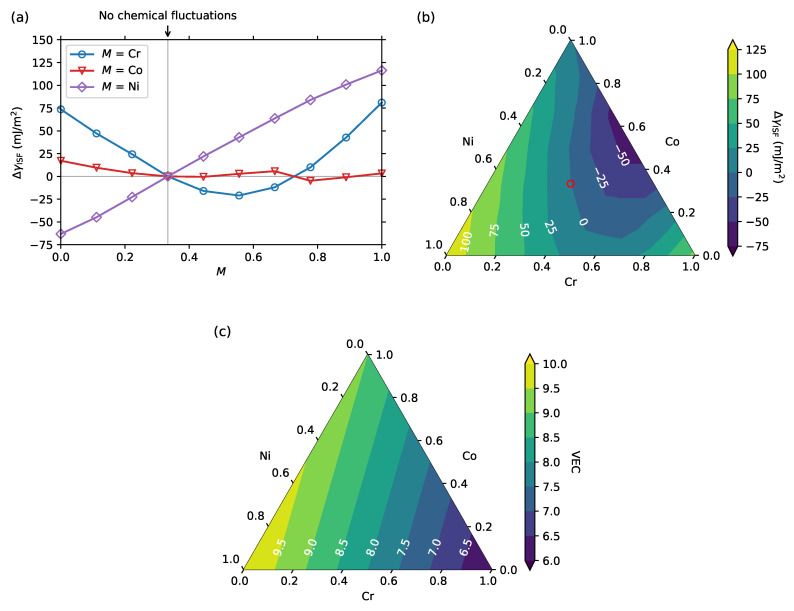
(**a**,**b**) Differences between the computed ISFEs of CrCoNi from those where no compositional fluctuation exists in the L1 layers. (**a**) Result as a function of the local concentration of the element *M* (*M* = Cr, Co, Ni) in the L1 layers. The other elements were kept equiatomic in the L1 layers. The vertical gray line indicates the point of the ideal solid solution, i.e., without chemical fluctuations near the SF; (**b**) Result as a function of the local composition ratio in the L1 layers. The red circle indicates the equiatomic composition ratio; (**c**) The valence electron concentration (VEC) as a function of the composition ratio.

We first discuss the impact of compositional fluctuations in the vicinity of the ISF for CrCoNi with increasing or decreasing individual elemental concentrations. The results are shown in Figure 2a.

The ISFE was found to increase monotonically with the increase of local Ni concentration close to the ISFs. This suggests that Ni segregation towards the ISF is thermodynamically limited and that there is a strong driving force to deplete the Ni concentration in the vicinity of the ISFs. This can be intuitively understood by the fact that pure Ni energetically prefers the fcc phase (groundstate of Ni), whereas in the vicinity of the ISF, the stacking order of the close-packed planes is similar to that of the hcp structure. If the layers close to the ISF are fully occupied by Ni, the ISFE increases by more than 100 mJ/m${}^{2}$, and when Ni is fully suppressed from the ISF, the ISFE decreases by more than 50 mJ/m${}^{2}$. This is consistent with previous computational results based on supercell models [24] in which the ISF with the local composition ratio of Cr${}_{8}$Co${}_{10}$Ni${}_{14}$ was 59 mJ/m${}^{2}$ higher than the ISF with the local composition ratio of Cr${}_{12}$Co${}_{10}$Ni${}_{10}$. It should be noted that the results in Ref. [24] were obtained based on non-spin-polarized calculations and another type of supercell approach (slab + vaccum layer), which prohibits a quantitative comparison.

The impact of Cr turned out to be somewhat more complex. The minimum ISFE was found at a Cr concentration of about 0.5, whereas the ISFE increased when the L1 layers were either highly occupied by Cr or mostly free from it. When Cr fully occupied the layers close to the ISF, the ISFE increased by more than 80 mJ/m${}^{2}$. A similar non-linear behavior of the ISFE was also found for the ferromagnetic (FM) fcc Cr${}_{x}$Co${}_{1-x}$ binary alloys by first-principles calculations [65]. This suggests that such a complex impact of Cr on the ISFE may be also found in other random 3d transition metal alloys.

When Co fully occupied the L1 layers, the ISFE did not change largely from when no chemical fluctuations existed, which suggests that Co in CrCoNi reveals a small fcc–hcp energy difference. This could be reasoned as follows. The groundstate of pure Co is the FM hcp phase, while with increasing temperature, it experiences both a structural transition to the fcc phase at around 700 K [66] and a magnetic transition at around 1400 K [66]. As found in previous works [67,68], magnetic fluctuations in Co contribute to the stability of the fcc phase at elevated temperatures. For example, in Ref. [68], the fcc–hcp energy difference of pure Co was found to be significantly reduced in the DLM state compared to the FM state. From the above considerations on pure Co, it can be intuited that the small ISFE change with respect to the local Co concentration close to the ISF is caused by the paramagnetic state of CrCoNi.

To further elucidate the impact of the non-linear trend of the compositional fluctuations for the ISFEs, we further explored the full compositional region for the L1 layers, as shown in Figure 2b. Overall, the ISFE varied in the range of approximately 180 mJ/m${}^{2}$, depending on the local composition ratio of the L1 layers. The strongest decrease in the ISFE of more than 60 mJ/m${}^{2}$ was found for nearly equiatomic Cr and Co occupying the L1 layers.

Large variation in the ISFE for CrCoNi was also found in previous computational works using supercell models with and without ISFs, where the computed ISFEs of CrCoNi were distributed in the range of 59 mJ/m${}^{2}$ [24], approximately 230 mJ/m${}^{2}$ [29], or 205 mJ/m${}^{2}$ [30], or with a standard deviation of approximately 110 mJ/m${}^{2}$ [26]. Note that the ISFE variation range in Ref. [24] was relatively small compared with that in Refs. [26,29,30]. This might be related to the limited set of considered configurations, or the usage of non-spin-polarized calculations and the suppression of energy fluctuations due to the magnetic degrees of freedom. This is consistent with the finding in Ref. [29], where the fluctuations of the ISFEs in CrMnFeCoNi were much smaller in the nonmagnetic state compared to the spin-polarized one.

As found in Figure 2a,b, the ISFE of CrCoNi varied non-linearly with respect to the local chemical composition close to the ISFs. This became even clearer by considering the VEC dependence on the ISFE, which is often employed to predict the phase stability of HEAs and CCAs [69,70]. The relations between the ISFE and the VEC near the ISFs for CrCoNi and CrFeCoNi have been discussed previously [24]. Figure 2c shows the VEC values for the same composition region as that in Figure 2b. By construction, VEC depends linearly on the chemical concentrations, while the trend does not match that of the actually computed ISFE, except for under Ni-rich conditions. This indicates that the VEC is, in general, not a sufficient quantitative descriptor for estimating the variation of ISFEs.

**Figure 3:**
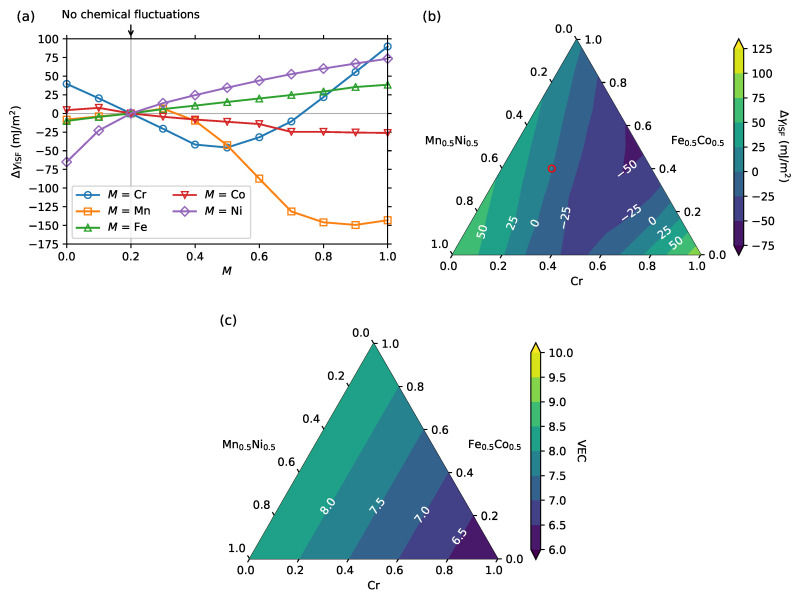
(**a**,**b**) Differences between the computed intrinsic SFE (ISFEs) of CrMnFeCoNi from those where no compositional fluctuation exists in the L1 layers. (**a**) Result as a function of the local concentration of the element *M* (*M* = Cr, Mn, Fe, Co, Ni) in the L1 layers. The other elements were kept equiatomic in the L1 layers. The vertical gray line indicates the point of the ideal solid solution, i.e., without chemical fluctuations near the SF; (**b**) Result as a function of the local composition ratio in the L1 layers in the pseudoternary region of Cr, Fe${}_{0.5}$Co${}_{0.5}$, and Mn${}_{0.5}$Ni${}_{0.5}$. The red circle indicates the equiatomic composition ratio; (**c**) The valence electron concentration (VEC) as a function of the composition ratio in the same pseudoternary region as that of (**b**).

We next considered the five-component fcc CrMnFeCoNi HEA, the so-called Cantor alloy. The impact of compositional fluctuations in the L1 layers on the computed ISFE are shown in Figure 3a. The ISFE depended on the local concentrations of Cr, Co, and Ni similarly, as found for CrCoNi. The ISFE monotonically increased with an increase in the local Ni concentration in the L1 layers. The ISFE also increased both when Cr nearly fully occupied the L1 layers and when it was nearly fully excluded in the L1 layers, while the ISFE decreased when the local Cr concentration in the L1 layers was around 0.5. Co showed a relatively small impact on the ISFE compared to the other elements also for CrMnFeCoNi with a slight decrease in the ISFE when fully occupying the L1 layers. The impact of Mn on the ISFE is clearly non-linear, as also found for Cr. When the local Mn concentration in the L1 layers was less than 0.4, the ISFE was hardly affected. In contrast, when the local Mn concentration in the L1 layers was larger than 0.4, the ISFE drastically decreased down to approximately 150 mJ/m${}^{2}$ with Mn fully occupying the L1 layers.

Recent experiments [63,71] have reported the phase decomposition of the CrMnFeCoNi alloy into body-centered cubic Cr, L1${}_{0}$ MnNi, and B2 FeCo after annealing at 450–500 ${}^{\circ }$C. To resolve the possible relationship between the precursor of the phase decomposition and the corresponding local chemical fluctuations near the ISFs, the ISFEs were also computed for the local composition ratios in the L1 layers in the pseudoternary region of Cr, Fe${}_{0.5}$Co${}_{0.5}$, and Mn${}_{0.5}$Ni${}_{0.5}$. The results are shown in Figure 3b. The ISFE increased with an increasing amount of Mn${}_{0.5}$Ni${}_{0.5}$, while the lowest ISFE was found when Mn${}_{0.5}$Ni${}_{0.5}$ was fully excluded from the L1 layers and replaced by a mixture of about 0.5 Cr and 0.5 Fe${}_{0.5}$Co${}_{0.5}$. This suggests that ISFs are suppressed in the presence of MnNi clusters. The VEC-derived linear dependencies are shown in Figure 3c and reveal once more that the VEC is, in general, not a good predictor of the nonlinear dependence of ISFE on local chemical fluctuations.

## 4. Conclusions

We investigated the impact of compositional fluctuations on the ISFEs for the fcc equiatomic CrCoNi and CrMnFeCoNi alloys by employing first-principles calculations by combining the supercell and CPA approaches. For both alloys, the ISFEs were found to vary within a range of more than 150 mJ/m${}^{2}$ depending on the local chemical environment in the vicinity of the ISFs. The chemical dependencies were shown to be highly non-linear and therewith, strongly deviated from linear Vegard’s law-like behavior. Cr caused non-linear behavior in CrCoNi. In the CrMnFeCoNi alloy, the presence of MnNi clusters could suppress ISFs.

The strong SFE dependence on the local chemical compositions in the vicinity of the SFs indicated, on the one hand, that the SFs in HEAs and CCAs may promote particular types of chemical segregations towards the SFs. On the other hand, if chemical fluctuations exist in HEAs and CCAs on a large enough scale, SFs are likely to occur in the local chemical environments with low SFEs. These potential behaviors further complicate the prediction of physical descriptors to determine deformation mechanisms in HEAs and CCAs compared to, e.g., unary metals or ordered alloys. The complexity might be further enhanced in the presence of chemical SRO, which impacts the probabilities of local chemical fluctuations compared to the ideal mixing state. At the same time, the revealed dependence of SFEs on local chemical fluctuations in the vicinity of SFs opens the route towards tuning alloy properties of HEAs and CCAs via controlling chemical fluctuations by tuning, e.g., the annealing conditions in the alloy processing route. Our results encourage further experimental analyses employing, e.g., transmission electron microscopy/energy-dispersive X-ray spectroscopy or atomic probe tomography to explore the role of chemical fluctuations in the vicinity of SFs in more detail.
